# Pseudohypoadrenocorticism in a Siberian Husky with* Trichuris vulpis* Infection

**DOI:** 10.1155/2019/3759683

**Published:** 2019-05-23

**Authors:** Stephanie Car, Catriona Croton, Mark Haworth

**Affiliations:** Small Animal Hospital, University of Queensland, Gatton 4343, Australia

## Abstract

An entire male Siberian Husky presented for diarrhoea, weakness, inappetence, and collapse following a six-day period of illness. On clinical examination the dog displayed vasoconstrictive circulatory shock, dehydration, and melena. Laboratory tests revealed a marked hyponatraemia, hyperkalaemia, and a decreased sodium/potassium ratio of ≤ 12.4. The baseline and poststimulation serum cortisol concentrations were markedly elevated following adrenocorticotropin hormone (ACTH) stimulation test, yielding 712 nmol/L and 706 nmol/L, respectively. The elevated cortisol concentration excluded hypoadrenocorticism. A concurrent* Trichuris vulpis* (whipworm) infection was also identified. The dog was treated with supportive care including fenbendazole and recovered uneventfully. The final diagnosis was* Trichuris vulpis* infection with secondary pseudohypoadrenocorticism. This case report further supports a previous observation that the Siberian Husky breed may have an increased sensitivity to infection with* Trichuris vulpis* and development of pseudohypoadrenocorticism.

## 1. Introduction

*Trichuris vulpis*, also known as the canine whipworm, is a nematode that inhabits the cecum and colon of dogs [1]. Dogs with whipworm infection can present with diarrhoea, inappetence or anorexia, dehydration, weight-loss, and weakness as well as hyponatraemia, hyperkalaemia, metabolic acidosis, and a decreased sodium/potassium (Na:K) ratio [2–7]. These findings resemble hypoadrenocorticism. However, this may be ruled out by an ACTH stimulation test which has previously shown normal to elevated basal cortisol concentrations and an appropriate adrenal response after stimulation [5]. While this has been reported previously in several breeds, Ruckstuhl et al. [7] described two such cases of Siberian Huskies that also developed severe electrolyte derangements including an extremely low Na:K ratio. This report illustrates a similar degree of electrolyte alterations in a Siberian Husky supporting the postulate asserted by Ruckstuhl et al. [7] that Siberian Huskies may be particularly susceptible to pseudohypoadrenocorticism secondary to T.* vulpis* infection.

## 2. Case History

A 7-year-old entire male Siberian Husky presented with a history of diarrhoea and lethargy for 1 week and inappetence for 4 days. Clinical examination at presentation showed vasoconstrictive circulatory shock, dehydration (10-12%), bradycardia (60 beats per minute), muscle weakness, and abdominal pain. The mucous membranes were pale, and the capillary refill time was 3 seconds. A worm was observed on rectal examination which was identified as* Trichuris vulpis *on microscopic inspection. Initial blood work showed an elevated serum lactate and a marked hyperkalaemia and hyponatraemia, with a potassium of 8.1 mmol/L (reference range: 3.4 – 4.9 mmol/L) and a sodium of less than 100 mmol/L (reference range: 135 – 153 mmol/L) (Table 1). The Na:K ratio was less than 12.4 (reference range: 27 - 38), although an exact value could not be determined as the serum sodium was below the limit of detection. A mild hypocalcaemia, hyperglycaemia, hyperphosphataemia, hypercholesterolaemia, metabolic acidosis, and a twofold increase in urea were also found. The serum creatinine concentration was not able to be determined initially and was repeated 4 hours after presentation and found to be within the reference interval. An ACTH stimulation test was performed in which 5.5 *μ*g/kg synthetic ACTH (tetracosactrin; Link Medical Products Pty Ltd., Warriewood, New South Wales, Australia) was administered intravenously. Serum samples were collected immediately prior to ACTH administration and one hour after administration.

Supportive treatment was initiated while awaiting ACTH stimulation results, with intravenous (IV) fluid therapy consisting of 0.45% NaCl and 2.5% glucose at 3 ml/kg/hr and concurrent compound sodium lactate solution at 3 ml/kg/hr. The serum sodium concentration was closely monitored, with the IV fluid therapy adjusted to ensure this did not rise faster than 10 mmol/L in the first 24 hours to avoid a demyelinating syndrome associated with rapid correction of hyponatraemia. Sodium administration was difficult to determine however as the initial serum sodium was unknown. In-house electrolyte monitoring 4 hours after commencement of treatment indicated an ongoing hyponatraemia (Table 1), and so the fluids were changed to 0.45% NaCl and 2.5% dextrose with 7% hypertonic saline added to make the total sodium concentration of 110 mmol/L. This IV fluid was infused at 10 ml/kg/hr. A serum chloride concentration was also performed at this time, which showed a hypochloraemia (84 mmol/L; reference range: 109-122 mmol/L). A corrected chloride calculation showed a normal relative concentration at 4 hours. Ten hours after admission, the dog's serum sodium had risen to 112 mmol/L, and the potassium fell from a peak of 8.4 mmol/L four hours after admission to 5.8 mmol/L. Additional treatment consisted of dexamethasone (Troy Laboratories Pty Ltd., Glendenning, New South Wales, Australia) at 0.1 mg/kg IV once. The patient showed a marked improvement in mentation and appetite had returned. Fenbendazole (Intervet Australia Pty Ltd., Bendigo East, Victoria, Australia) at 50 mg/kg orally once a day for three doses was also administered.

ACTH stimulation test results revealed that both the baseline and post ACTH stimulation serum cortisol were markedly elevated; the baseline serum cortisol was more than a sevenfold increase of the upper limit of the reference range at 712 nmol/L (reference range: 30-100 nmol/L). Poststimulation showed no increase in the serum cortisol concentration (Table 2).

Over the following three days of hospitalisation, electrolytes were frequently monitored. Fluid therapy was adjusted as required to restore serum sodium to the normal range no faster than approximately 0.5 mmol/L/hr. Watery brown diarrhoea continued throughout the hospital stay and* Trichuris vulpis *infection was confirmed by faecal examination and float with observation of a large number of eggs. Although electrolyte derangements had normalised by discharge, the serum sodium had only just reached the normal reference interval at 135 mmol/L. The hyperkalaemia had resolved, and serum potassium was 4.6 mmol/L with a Na:K ratio which was 29 (reference range: 27-38). The patient was discharged with instructions to administer monthly treatment against* T*.* vulpis* for 6 months and every 3 months thereafter.

## 3. Discussion

This case report details the development of a pseudohypoadrenocorticism in a Siberian Husky with chronic diarrhoea as a result of* T*.* vulpis* infection. The severity of decrease in the Na:K ratio mirrors the findings of two Siberian Huskies reported in a previous paper [7]. As a known cause of pseudohypoadrenocorticism it has been postulated that* T. vulpis* may cause more profound clinical signs in this breed, or this breed may be more prone to a severe infection of this parasite.

*Trichuris vulpis* infection was diagnosed by microscopic examination of the worm itself and faecal flotation. The worm tunnels through the mucosa of the caecum and large intestine using a stylet projection from its oral cavity to lacerate blood vessels and generate pools of fluid and blood to feed off [9]. Hence gastrointestinal signs such as diarrhoea, vomiting, and anorexia are common and lead to hypovolaemia and dehydration. Despite gastrointestinal fluid losses contributing to decrease circulating volume, the dog was found to have a relative bradycardia of 60 beats per minute which would certainly have contributed to decrease oxygen delivery and tissue ischaemia. This bradycardia was attributed to the hyperkalaemia.

Hyponatraemia and hypochloraemia are a sequela of the fluid losses which are usually isotonic. Subsequent replacement of this isotonic loss by drinking water yields a dilutional hyponatraemia. This is substantiated by the corrected chloride as the normal result suggests a free water gain causing hyponatraemia. A study examining serum aldosterone in 5 dogs with Trichuriasis-associated pseudohypoadrenocorticism demonstrated hyponatraemia and hyperkalaemia was not a result of selective aldosterone deficiency [5]. Hypovolaemia due to gastrointestinal fluid losses results in decreased tissue perfusion resulting in decreased distal tubular renal flow which has been suggested as a possible caused of hyperkalaemia [6]. Metabolic acidosis as a result of bicarbonate losses from diarrhoea and anaerobic metabolism has also been suggested as a cause for hyperkalaemia as a result of cellular translocation from the intracellular space into the extracellular space [8]. The dog reported here displayed a mild metabolic acidosis with minimal decrease in serum bicarbonate and base excess making the latter postulate for hyperkalaemia unlikely. The resultant hyponatraemia and hyperkalaemia can be severe, and the clinical picture can easily be mistaken for hypoadrenocorticism.

**Figure 1:**
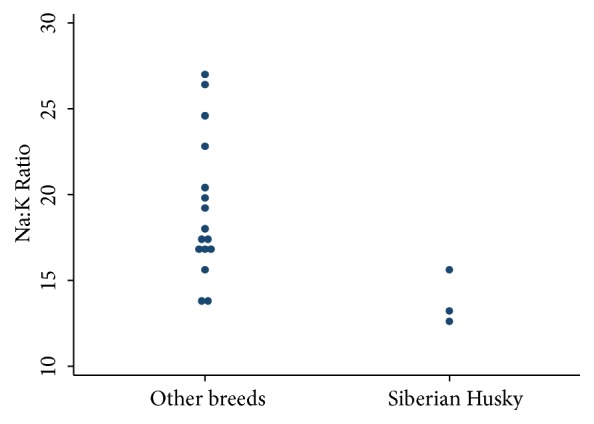
Dot plot comparing the Na:K Ratio for Siberian Huskies (n=3) to other breeds of dogs with Trichuriasis-associated pseudohypoadrenocorticism (n=16) reported in the literature [3, 5–8]. Analysed using Stata v14 (StataCorp LP, College Station, Texas).

In this case report, the Siberian Husky had a severe hyperkalaemia and hyponatraemia with a profoundly low Na:K ratio of less than 12.4. These changes are similar to those reported in two Siberian Huskies reported by Ruckstuhl et al. [7], who also described Siberian Huskies with unusually low Na:K ratios (13.2 and 15.4). Dibartola et al. [8] first described pseudohypoadrenocorticism in dogs with primary gastrointestinal disease. Seven dogs in this study with gastrointestinal signs had abnormal Na:K ratios attributed to* Trichuris vulpis* infection, and all but one with concurrent ancyclostomiasis infection were above 15 [8]. In addition, a study by Graves et al. found five dogs with Trichuriasis-associated pseudohypoadrenocorticism had a Na:K ratio above 15 [5]. In both of these studies the dogs were of varied breeds and none were noted to be Siberian Huskies. Another study by Malik et al. of Trichuriasis-associated pseudohypoadrenocorticism in three dogs reported two Na:K ratios of 17 and one of 14 [3]. None of these dogs were Huskies. Despite the small number of cases, it is possible that Siberian Huskies are overrepresented and have unusually low Na:K ratios associated with pseudohypoadrenocorticism from whipworm infection (Figure 1). According to Roth and Tyler [10], the most common cause of a Na:K ratio between 15 and 24 is renal or urinary tract disease, but if the Na:K ratio is markedly decreased (<15), this was exclusively due to true hypoadrenocorticism. This finding is also supported by Nielsen et al., where ratios below 17 were exclusively due to hypoadrenocorticism [11]. Another possibility for the magnitude of decrease in the Na:K ratio could relate to an exaggerated increase in serum potassium. The Asian breeds such as the Japanese Shiba and Akita dogs have been demonstrated to have high erythrocyte potassium concentrations [12, 13]. These breeds are not recommended as blood donors due to the risk of haemolysis and resultant hyperkalaemia. While unlikely, the Asian origin of this Siberian Husky could be considered a risk for pseudohyperkalaemia if any haemolysis in the sample analysed occurred. This would magnify the reduction in the Na:K ratio. However, the initial serum sodium concentration was below the limit of detection and the initial Na:K ratio was not able to be calculated. The two Siberian Huskies reported by Ruckstuhl et al., and the Siberian Husky reported here, all exhibited Na:K ratios under 15.4. The cause for this marked alteration compared to other breeds remains unknown. It is possible that Huskies may exhibit an increased sensitivity to whipworm infection or the dog reported here may have had an extreme worm burden.

The ACTH stimulation test allowed differentiation of pseudohypoadrenocorticism from true hypoadrenocorticism, as the baseline and poststimulation serum cortisol concentrations were markedly elevated following 6 days of illness (Table 2). These results are likely a response to the stress associated with the underlying disease of whipworm infection with secondary circulatory shock and dehydration. Several studies have examined the stress response in critically ill dogs and demonstrated that an exaggerated basal cortisol concentration was negatively associated with survival [14–16]. Further, the normal physiological response mounted by some patients may be insufficient for the degree of illness, and the adrenal gland may be unable to meet the demands of ongoing stress [17]. This dog demonstrated a very high basal cortisol concentration and no reserve capacity in the adrenal response to an ACTH challenge indicating the severity of the infection associated with* T. vulpis*.

In conclusion, this report details a Siberian Husky with marked laboratory alterations consistent with pseudohypoadrenocorticism secondary to* T. vulpis* infection. This report presents new evidence to support a previous hypothesis this breed may have an increased sensitivity to this parasite as demonstrated by very low Na:K ratio, usually only seen in true hypoadrenocorticism [18]. However, a larger number of cases would need to be evaluated which would be difficult given the rarity of pseudohypoadrenocorticism.

## Figures and Tables

**Table 1:** Initial blood results after admission and treatment (venous).

Parameter	Unit	Value (Initial)	Value (4 hours)	Value (10 hours)	Range
HCT	%	48	43	34	36 - 55
TP	g/L	71			50-72
pH		7.25	7.29	7.32	7.35 – 7.44
pCO_2_	mmHg	40	40	38	33 – 41
pO_2_	mmHg	21	30	32	
Na^+^	mmol/L	< 100	108	112	135 – 153
K^+^	mmol/L	8.1	8.4	5.8	3.4 – 4.9
Na:K		< 12.4	12.9	19.3	27-38
Cl^−^	mmol/L		84	97	105-116
Corrected Cl-	mmol/L		114	126	105-116
Ca^++^	mmol/L	0.91	0.92	0.99	1.12 – 1.4
Gluc	mmol/L	10.4	9.9	7.3	3.3 – 6.8
Lac	mmol/L	3.2	1.4	1.2	< 2.0
HC0_3_^−^	mmol/L	18.0	19.2	19.6	20.8-24.2
BEecf	mmol/L	-8.8	-6.9	-6.0	-1.2 ± 1.1
CREAT	*μ*mol/L	No result		37	44 -159
UREA	mmol/L	20.1			2.5 - 9.6
PHOS	mmol/L	2.63			0.81- 2.21
ALB	g/L	30			23 – 40
GLOB	g/L	41			25 – 45
ALT	U/L	58			10 – 125
ALKP	U/L	48			23 – 212
GGT	U/L	0			0 –11
TBIL	*μ*mol/L	13			0 – 15
CHOL	mmol/L	9.35			2.84 – 8.26

HCT: haematocrit, TP: total protein, pCO_2_: partial pressure of carbon dioxide, pO_2_: partial pressure of oxygen, Na^+^: sodium, K^+^: potassium, Cl^−^: chloride, Ca^++^: ionized calcium, Gluc: glucose, Lac: lactate, HCO_3_^−^: bicarbonate, BE: base excess, CREAT: creatinine, PHOS: phosphorus, ALB: albumin, GLOB: globulins, ALT: alanine aminotransferase, ALKP: alkaline phosphatase, GGT: gamma glutamyltransferase, TBIL: total bilirubin, and CHOL: cholesterol.

**Table 2:** Results of the ACTH Stimulation Test^*∗*^.

Test	Result	SI Units	Range
Cortisol (baseline)	712	nmol/L	30 – 100
Post ACTH Cortisol	706	nmol/L	220 – 550

ACTH: adrenocorticotropic hormone. ^*∗*^Cortisol assay performed on Immulite 1000.
